# A newborn with convulsions 12 days after birth was misdiagnosed as neonatal intracranial hemorrhage: Case report

**DOI:** 10.1097/MD.0000000000036675

**Published:** 2023-12-29

**Authors:** Yuanyuan Che, Jianmin Zhong, Yong Chen, Jihua Xie, Ruiyan Wang, Yuxin Xu, Jian Zha, Miao Zeng, Hui Chen

**Affiliations:** a Department of Neurology, Children’s Hospital of Jiangxi Province, Nanchang, China.

**Keywords:** intracranial hemorrhage, misdiagnosed, newborn, seizure, tuberous sclerosis complex

## Abstract

**Introduction:**

Cases with early diagnosis of neonatal tuberous sclerosis syndrome (TSC) are relatively seldom seen, and misdiagnosis of intracranial hemorrhage is even more rare. We retrospectively analyzed the clinical data of a case of neonatal tuberous sclerosis with atypical early symptoms and misdiagnosed as more common intracranial hemorrhage of the newborn.

**Patient concerns:**

The child was female and had no obvious cause of convulsion 12 days after birth. The local hospital was initially diagnosed as “neonatal intracranial hemorrhage, congenital heart disease,” and still had convulsions after 5 days of treatment, so it was transferred to neonatal intensive care unit of our hospital.

**Diagnosis:**

After admission, cardiac color ultrasound, magnetic resonance imaging, and electroencephalogram were performed, and TSC was diagnosed in combination with clinical symptoms. However, no known pathogenic mutations such as TSC1 and TSC2 were detected by peripheral blood whole exon sequencing.

**Intervention:**

After a clear diagnosis, sirolimus, and vigabatrin were given. But there were still convulsions. Topiramate, valproic acid, and oxcarbazepine were successively added to the outpatient department for antiepileptic treatment, and vigabatrin gradually decreased.

**Outcome:**

Up to now, although the seizures have decreased, they have not been completely controlled.

**Conclusions:**

The TSC of neonatal tuberous sclerosis is different from that of older children. It is usually characterized by respiratory distress and arrhythmia, and may be accompanied by convulsions, but the activity between attacks is normal. However, neonatal intracranial hemorrhage can be caused by premature delivery, birth injury, hypoxia, etc. Its characteristics are acute onset, severe illness, and rapid progression. Consequently, the diagnosis of these 2 diseases should not only be based on medical imaging, but also be combined with their clinical characteristics. When the imaging features are inconsistent with the clinical diagnosis, a comprehensive evaluation should be made again. The timing and pattern of onset of neonatal convulsions can help in differential diagnosis. If there is cardiac rhabdomyoma, subependymal or cortical nodule, skin low melanoma, etc, the possibility of neonatal TSC should be considered, and the diagnosis should be made according to its diagnostic criteria to avoid or reduce misdiagnosis.

## 1. Introduction

Tuberous sclerosis complex is an autosomal dominant neurocutaneous syndrome with incomplete penetrance. It is mainly caused by TSC1 and TSC2 gene mutations, but 15% to 20% of them are still undetectable.^[1]^ The incidence rate among the surviving newborns is 1/15,400 to 1/5800.^[2]^ The clinical manifestations of different age groups are very different.^[3]^ The typical features are hamartomas involving multiple systems such as brain, heart, kidney, skin, and eyes. The central nervous system is most often affected. The early symptoms are not typical, which can easily lead to misdiagnosis as other diseases. Now, we report a case of neonatal tuberous sclerosis syndrome (TSC), which was initially diagnosed as intracranial hemorrhage, in order to improve our understanding of neonatal TSC.

## 2. Case presentation

**Figure 1.**
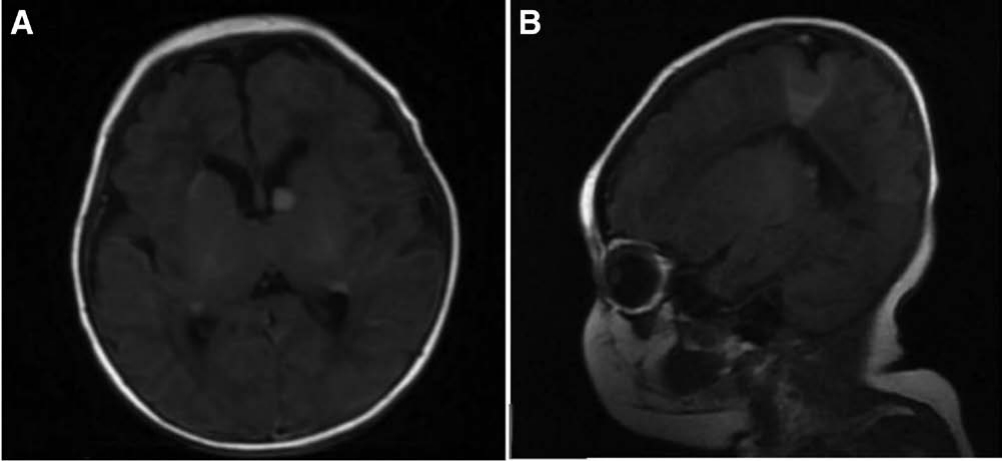
The head MRI showed that there were many spot-shaped abnormal signal shadows under the ependymal of bilateral lateral ventricles, T1 was high, T2 was low; the left parietal cortex is locally thickened, showing patchy T1 uneven high signal, and DWi slightly high signal.

**Figure 2.**
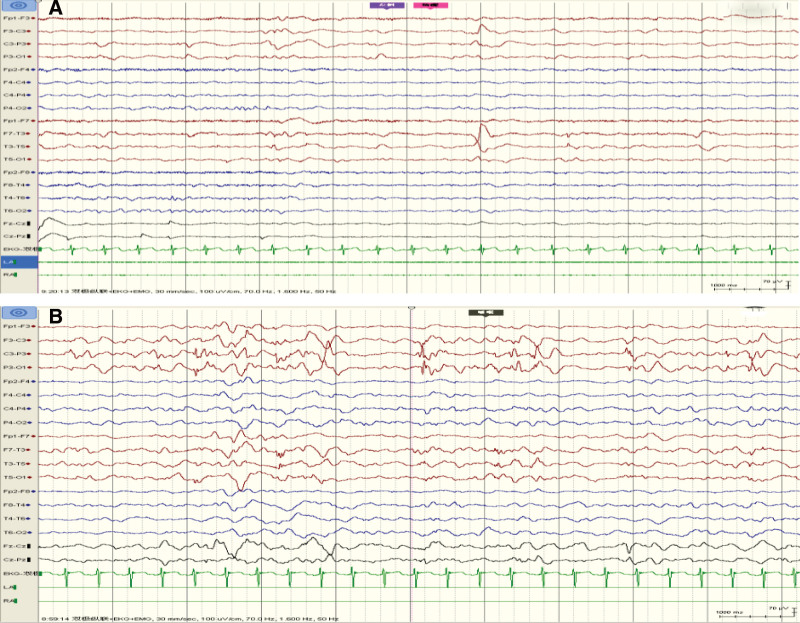
The long range video EEG of the newborn with 23 days old (postmenstrual age was 41 weeks and 6 days) showed that a large number of left hemisphere mainly distributed multifocal sharp waves and sharp and slow waves during the interval.

**Figure 3.**
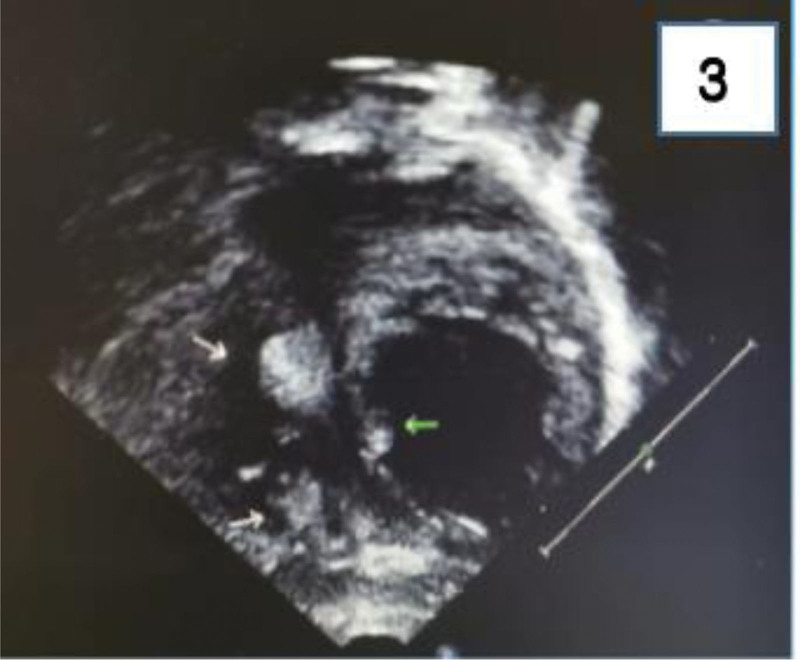
Echo enhancement of left and right ventricles and left ventricular outflow tract (multiple rhabdomyomas are possible) is shown by color Doppler echocardiography.

The child, female, 20 days old, was admitted with 8 days of recurrent seizures as the main complaint. The baby was born at the first delivery of the third fetus, and was delivered by cesarean section at 38 weeks and 4 days of gestation. She had no history of intrauterine distress and birth asphyxia, and her birth weight was 3650 g. The mother received progesterone for 2 months from the second month of pregnancy because of low progesterone. The first 2 fetuses were aborted at 2 months of gestation. The parents were not close relatives and denied the family history of hereditary diseases. From the 12th day of life, there was no obvious cause of convulsion, which was manifested as frequent blinking of the left eye, twitching of the left corner of the mouth, not involving the limbs, lasting for more than 10 seconds. After relief, mental activity remained normal, and there was no fever, vomiting, cough, runny nose, and foam sputum at the mouth. The head computed tomography (CT) of the local hospital showed multiple high-density shadows in both hemispheres of the brain and under the ependymal membrane of both ventricles. The color Doppler ultrasound of the heart showed hyperechoic masses in the left ventricular outflow tract. It was intended to be “intracranial hemorrhage of the newborn, congenital heart disease.” After 5 days of treatment with anti-infection, hemostasis and fluid replacement, it did not improve and still had convulsions, so it was transferred to neonatal intensive care unit of our hospital. During the course of the disease, the child had no fever, shortness of breath and cyanosis, and the stool and urine were normal. The physical examination on admission showed poor response, the heart heard grade II/6 murmur, the left 6 fingers were deformed, and no obvious abnormality was found. The admission diagnosis was “neonatal convulsion, central nervous system infection, neonatal intracranial hemorrhage, congenital heart disease, polydactyly.” After admission, cerebrospinal fluid routine and biochemistry were normal, and no bacteria were cultured. Hemochlamydia IgM was positive. Head magnetic resonance imaging (MRI) displayed that patchy T1 slightly high signal shadow, T2 slightly low signal shadow, localized thickening of left parietal cortex, patchy uneven high signal, diffusion weighted imaging slightly high signal were scattered in both cerebral hemispheres. There are many spot-shaped abnormal signal shadows in the subependymal part of the bilayer lateral ventricle, T1 iso-signal, T2 hypo-signal shadows. The sigmoid sinus of the left transverse sinus is not clear. It suggests tuberous sclerosis; focal cortical dysplasia in the left parietal lobe (Fig. 1). Three-hour video electroencephalography (EEG) demonstrated sharp waves in the left hemisphere and the right frontal pole, frontal, and temporal regions. The left hemisphere was marked (Fig. 2).After admission, renal color ultrasound showed multiple anechoic areas in both kidneys, about 19 * 13 mm, indicating multiple cysts in both kidneys. Echocardiography in our hospital revealed enhanced echogenicity of left and right ventricles and left ventricular outflow tract. The diameter of left ventricular outflow tract was slightly narrowed, suggesting that multiple rhabdomyomas might occur (Fig. 3). During the consultation of neurology department, several depigmentation spots were found on the body, several on the left front chest (0.3 cm × 0.5 cm–0.4 cm × 1 cm), one on the right waist (2.5 cm × 1 cm) and one on the perineum (0.5 cm × 0.5 cm). It was comprehensively diagnosed as tuberous sclerosis. On the sixth day after admission, sirolimus was given orally 1 mg/(m^2^·d) qd and vigabatrin 25 mg/kg/d bid. No known pathogenic mutations such as TSC1 and TSC2 were detected using the full exon sequencing method in peripheral blood samples collected from the child. After 1 week of treatment, there was no significant reduction in seizures. The 3-hour video EEG showed that the left hemisphere had multiple lesions and the right occipital and temporal areas had sharp waves and slow waves. The left hemisphere was significant. When the dosage of vigabatrin was increased to 50 mg/kg·d bid, the seizures were reduced, but there were still seizures. The parents requested that the treatment plan be adjusted at the discharge follow-up clinic.

**Figure 4.**
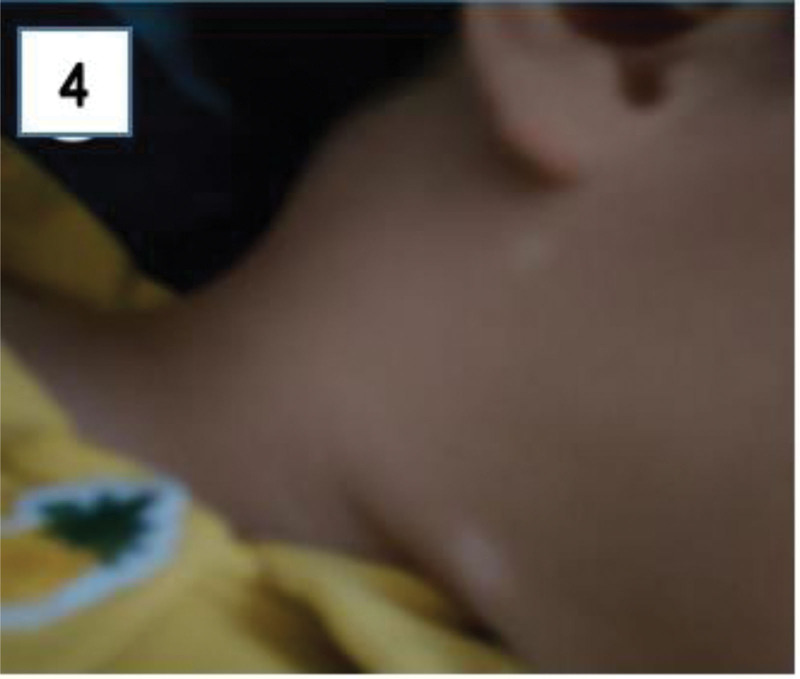
Pigment loss spots can be seen on the right face and mandible.

**Figure 5.**
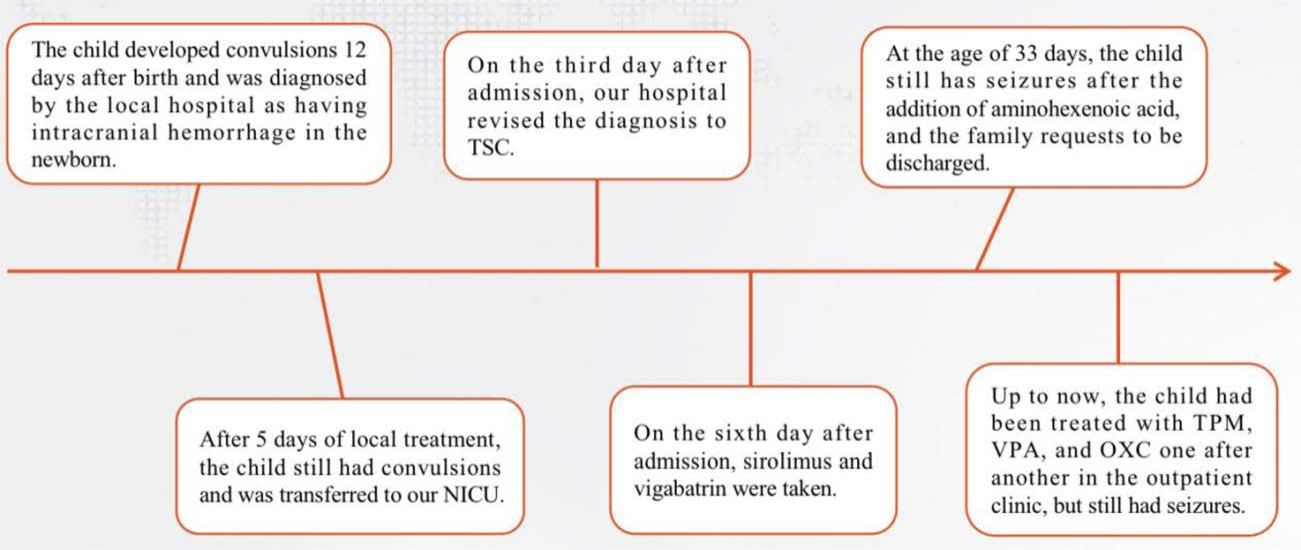
This is a flowchart of the diagnosis and treatment of the child.

After discharge, vigabatrin was gradually titrated to 120 mg/kg/d, bid; 1% sirolimus oral solution 1 mg/(m^2^·d), qd. The attack was reduced by more than 50% compared with the previous, but there were still convulsions, and no attack occurred for up to 2 days. At the age of 3 months and 18 days, the 0 to 1-year-old neuromotor examination scale and the Denver developmental screening tests screening scale lagged behind the children of the same age, and Gesell developmental quotient of large motor, fine motor and individual-social development was equivalent to that of children of 2 months. Four days later, the 3-hour video EEG showed that the left hemisphere had multifocal sharp and slow waves; 2 left initial focal seizures were detected during waking period. At 4 months, sirolimus oral solution was 1 mg/(m^2^·d), qd, and topiramate, valproic acid, and oxcarbazepine were successively added to the outpatient department for antiepileptic treatment, and vigabatrin gradually decreased to 55 mg/kg/d, bid. When returning to the outpatient clinic for follow-up, her parents often discussed the condition and efficacy with other parents of TSC patients. Up to now, although the seizures have decreased, they have not been completely controlled. There are more than 10 spots of pigmentation loss in the neck, back, chest, and abdomen, 0.5 cm × 0.5 cm to 3 cm × 2 cm, some from the original 0.3 cm × 0.5 cm to 1.5 cm × 1 cm (Fig. 4).The entire diagnosis and treatment process of this child is shown in Figure 5 below.

This study obtained the informed consent of the child’s guardian and was approved by the Medical Ethics Committee of Jiangxi Children’s Hospital.

## 3. Discussion and conclusions

Neonatal seizures are common manifestations of neurological dysfunction in newborns, and their causes include hypoxic ischemic encephalopathy (HIE), neonatal stroke, intracranial hemorrhage, intracranial infection, genetic, and metabolic diseases, congenital malformations, and unexplained convulsions. The etiology of neonatal seizures is mostly acute symptomatic,^[4]^ mostly occurring within 1 week after birth. Many studies have shown that HIE accounts for 60% to 65% of children with seizures within 24 hours after birth, and seizures caused by HIE mostly occur within 3 days after birth. Convulsions occurring within 72 hours after birth are mainly acute symptoms, which may be related to stroke or brain malformations, suppurative meningitis, intraventricular hemorrhage in preterm infants, withdrawal syndrome, and metabolic disorders. Genetically related seizures, such as benign familial seizures in newborns, are commonly seen at the end of the first week of life. The types of seizures in newborns include microminiature, clonic type, tonic type, myoclonic type, spastic type, and so on. The forms of seizures are variable and difficult to identify. Clinical seizure types can provide evidence for the etiology of convulsions, such as metabolic disorders can cause myoclonic seizures, stroke, intracranial hemorrhage, focal cortical dysplasia that can cause focal clonic seizures, and children with congenital epilepsy syndrome exhibit tonic seizures.^[5]^ The child in our case developed convulsions on the 12th day after birth, which is a relatively rare neonatal TSC.

TSC is a multi-system autosomal dominant genetic disease characterized by multiple organ benign tumors, which may involve the skin, brain, kidney, lung, and heart, and occasionally may become malignant. The clinical manifestations are different in different ages, and the organs and severity of hamartoma are also different. The difference of clinical manifestations makes the diagnosis of TSC in infants, especially newborns, very challenging. Mutation of TSC1 or TSC2 gene can cause abnormal activation of mammalian target of rapamycin pathway, which leads to TSC caused by nerve cell growth, proliferation, abnormal cell morphology, and abnormal cortical development. However, as reported by Peron et al, at least 10% of TSC cases could not detect gene mutations.^[6]^ No pathogenic mutation was found in this case through gene detection. There may be chimerism, microdeletion, microduplication, deep intron mutation or other unknown mutation modes. Therefore, when the clinical manifestation is atypical and the gene test is negative, it is easy to cause misdiagnosis, missed diagnosis or delayed diagnosis.

The clinical manifestations of TSC patients differ greatly from that of children in the neonatal period, and the neonatal period is usually atypical, which easily leads to misdiagnosis and missed diagnosis. Isaacs^[7]^ carried out a retrospective study on 70 cases of TSC, of which 27 cases of neonatal patients had the initial main symptoms of respiratory distress, arrhythmia, heart murmur, and cardiac enlargement, which were mostly caused by cardiac rhabdomyoma, but also could occur in congenital heart disease. Since the incidence rate of the latter was as high as 8% to 9%,^[8]^ far higher than that of TSC, it was easy to be misdiagnosed as congenital heart disease. Two thirds of newborn patients have at least 1 rhabdomyoma, which can interfere with the ventricular blood flow and conduction system. In severe cases, it can cause intractable arrhythmia and cardiac failure, and is often the main cause of death in neonatal period. In this case, the left and right ventricles and the left ventricular outflow tract were rhabdomyomas, which led to the narrowing of the left ventricular outflow tract inner diameter and blocked blood flow. The heart heard grade II/6 murmur, which further confirmed the above view. In addition, in 27 cases of neonatal TSC reported in the literature, ≤1% of the children had skin damage and retinopathy. In childhood, almost all TSC patients will have skin symptoms. These skin symptoms usually occur at different times.^[9]^ However, in the 8 cases of neonatal TSC retrospectively analyzed by Zheng Xu et al,^[10]^ 75% of the patients had skin changes during the neonatal period, including 5 cases of depigmentation, 2 cases of angiofibroma, and 1 case of sharkskin plaques. There are great differences between domestic and foreign studies on skin damage, and the reasons are unknown. Due to the small number of cases, there may be case-selective bias. In this case, there is also depigmentation in the neonatal period, and it appears continuously with the increase of age, showing an increasing trend. However, the neonatal body surface area is small, the degree is light, the scope is small, and the physical examination is easy to be ignored.80% to 90% of children with TSC in childhood have seizures, usually within 1 year after birth; however, TSC in neonatal period is only 5%, which is often associated with large cortical malformation and subependymal giant cell astrocytoma.^[11]^ Kotulska et al^[12]^ reviewed 421 cases of TSC, 21 cases (5.7%) had seizures in neonatal period. Among the 21 children, 90.5% of them started with focal seizures, 47.6% with tonic-clonic seizures, 33.3% with clonic seizures, 28.6% with epileptic seizures, 23.8% with tonic seizures, 2 with dystonia, 1 with myoclonic seizures and 1 with eyelid myoclonic seizures. Hypoxic–ischemic encephalopathy, stroke, and infection are the most common neonatal convulsions,^[13]^ while neonatal TSC seizures are rare and lack of characteristics, which may also be one of the reasons for misdiagnosis or missed diagnosis of neonatal TSC. In 21 cases of EEG monitoring, 71.2% of them had abnormal background activity, 90.5% had sharp wave and/or spike wave during the interval, and 33.3% had clinical electrical seizures.^[12]^ This child developed seizures on the 12th day after birth. Video electroencephalogram monitoring was conducted for 3 hours on the 23rd and 30th days after birth, and no seizure was detected. However, a large number of multifocal sharp waves and sharp and slow waves mainly in the left hemisphere were seen during the interval. The origin of TSC seizures is not fully understood. The neuropathological basis of epilepsy is due to cortical nodules, but more and more evidence shows that it originates from the cortex around the nodules.^[14]^ In the brain of TSC patients, the expression of γ-aminobutyric acid type A receptor (GABAAR) subunit (including GABAAR α 1, GABAAR α 4, and GABAAR α 5) is down-regulated, which may provide a biological basis for the occurrence of early seizures and encephalopathy.^[15]^ In 2021, the International TSC Consensus Conference developed a new version of the diagnosis and treatment guidelines.^[16]^ The main features of this case include cortical nodules, subependymal nodules, cardiac rhabdomyoma, and more than 3 depigmentation of pigment. It can be clearly diagnosed if it meets 2 or more main features. Intracranial hemorrhage is more common in neonates, and the incidence of full-term newborns is 27% to 26%,^[17]^ the incidence of gestational age <32 weeks and extremely low birth weight can be as high as about 50%,^[18]^ far higher than TSC. Subarachnoid hemorrhage and periventricular–intraventricular hemorrhage are the most common intracranial hemorrhage in neonates, followed by subdural hemorrhage and cerebral parenchymal hemorrhage, and intracranial hemorrhage is mostly progressive. Hemorrhagic foci are mostly high density shadows in CT examination, and the edge of subependymal hemorrhage is relatively clear.^[19]^ In CT scan, the subependymal noncalcified nodules usually appear as isodense or slightly high-density shadows, and the cortical and subcortical nodules often appear as isodense or low-density shadows,^[20]^ sometimes easily confused with intracranial hemorrhage. Head MRI showed T1 isosignal or low signal and T2 high signal within 3 days of hemorrhage, and then changed into T1 high signal and T2 low signal after 3 days of hemorrhage, with strong specificity and high sensitivity. The MRI of this patient was performed 3 days after the course of the disease. The patchy T1 slightly high signal shadow, diffusion weighted imaging slightly high signal shadow, multiple dots of T1 and other signals under the ependymal membrane of the bilateral ventricles, and T2 low signal shadow were scattered in both cerebral hemispheres. The bleeding focus is generally low signal shadow in susceptibility weighted imaging imaging, and the image change observation of the bleeding focus can also help to judge.

The first choice for the treatment of TSC-related epilepsy is drug therapy. In 2012, the international TSC consensus recommended the targeted treatment of the mammalian target of rapamycin inhibitor rapamycin and its derivative everolimus, which can better control the seizures and is relatively safe. At present, everolimus has been approved for use. 40% to 80% of cases are effective, and the effective rate of rapamycin can reach about 70%.^[21]^Henske et al^[9]^ pointed out that vigabatrin is recommended as the first-line treatment drug for TSC-related epilepsy by European and international experts. At present, there is no large sample study on the treatment of TSC-related epilepsy in the neonatal period, and the effectiveness and safety of the drug need to be further verified. Phenobarbital drugs are mostly used for the treatment of neonatal anticonvulsant, and drugs such as vigabatrin are still recurrent and difficult to control.^[11]^ A multicenter study aimed at 94 infants with TSC, including newborns, who had no seizures. The results showed that preventive treatment with vigabatrin was safe and effective, which could change the natural history of seizures and reduce the risk and severity of epilepsy.^[22]^ When drug treatment is not well controlled, children with TSC can also consider focus surgery, vagus nerve stimulation and ketogenic diet.TSC surgery in childhood is mostly applicable to drug-refractory epileptogenic nodules with consistent symptoms, imaging and electrophysiology, but cortical nodules are relatively stable until 1 year old, so surgical treatment is usually considered after 1 year old.^[9]^ For the neonates with subependymal giant cell astrocytoma, dysplasia with transmantle sign, especially hemimegacephaly, whether or not to operate and the timing of the operation for those whose seizures are difficult to control still need further study. A TSC child with megagyrus underwent functional hemispherectomy 7 weeks after birth, and there was no attack 8 years after operation.^[11]^ After the diagnosis, this patient was given sirolimus and vigabatrin, but the seizures were not controlled. After topiramate was added, there were still seizures, which was drug-refractory epilepsy, consistent with 70% of TSC-related epilepsy reported in the literature as drug-refractory epilepsy.^[23]^

Newborn intracranial hemorrhage is more common than TSC. Newborn intracranial hemorrhage often has definite causes such as birth injury or hypoxic brain injury. Most of them are premature infants. Periventricular and intraventricular hemorrhage are the most common. The onset of the disease is acute, the condition is serious, the mortality is high, and often accompanied by convulsions, coma, increased intracranial pressure, changes in pupil, respiration and muscle tone. However, neonatal TSC mostly has no clear cause, with respiratory distress and arrhythmia as the main clinical manifestations, and a few can be accompanied by convulsions, with normal activities during the interval. Therefore, for the case of intracranial hemorrhage of the newborn to be diagnosed, the diagnosis cannot be made only by imaging examination. When it is inconsistent with the clinical manifestations, the clinical and imaging evaluation should be carried out again. If cardiac rhabdomyoma, subependymal or cortical nodule, depigmentation of skin, etc are found in the examination, the possibility of neonatal TSC should be considered and diagnosed according to the corresponding diagnostic criteria to avoid or reduce misdiagnosis.

Typical TSC is not difficult to diagnose clinically. Due to the lack of obvious characteristics of TSC during the neonatal period, it is easy to miss and misdiagnose. The timing and pattern of onset of neonatal convulsions can help in differential diagnosis. Early detection of lesions depends on prenatal and postpartum cardiac ultrasound and cranial imaging. As the disease progresses, when the clinical manifestations contradict the imaging findings, a comprehensive evaluation should be conducted again. A negative result of gene mutation testing does not preclude the diagnosis of TSC. It should also be emphasized that the early diagnosis of tuberous sclerosis is of great significance in guiding the treatment of the disease.

## Author contributions

**Conceptualization:** Yuanyuan Che, Jianmin Zhong, Yong Chen.

**Data curation:** Yuanyuan Che, Ruiyan Wang.

**Formal analysis:** Jihua Xie, Yuxin Xu.

**Methodology:** Jianmin Zhong, Yong Chen, Yuxin Xu, Jian Zha, Hui Chen.

**Resources:** Miao Zeng.

**Software:** Jian Zha, Hui Chen.

**Supervision:** Yong Chen, Jihua Xie, Ruiyan Wang.

**Writing – original draft:** Yuanyuan Che.

**Writing – review & editing:** Jianmin Zhong.
